# Weighing In on mTOR Complex 2 Signaling: The Expanding Role in Cell Metabolism

**DOI:** 10.1155/2018/7838647

**Published:** 2018-10-30

**Authors:** Yongting Luo, Wenyi Xu, Guannan Li, Wei Cui

**Affiliations:** ^1^Beijing Advanced Innovation Center for Food Nutrition and Human Health, China Agricultural University, Beijing 100193, China; ^2^Institute of Reproductive and Developmental Biology, Department of Surgery and Cancer, Imperial College London, Du Cane Road, London W12 0NN, UK

## Abstract

In all eukaryotes, the mechanistic target of rapamycin (mTOR) signaling emerges as a master regulator of homeostasis, which integrates environmental inputs, including nutrients, energy, and growth factors, to regulate many fundamental cellular processes such as cell growth and metabolism. mTOR signaling functions through two structurally and functionally distinct complexes, mTOR complex 1 (mTORC1) and mTOR complex 2 (mTORC2), which correspond to two major branches of signal output. While mTORC1 is well characterized for its structure, regulation, and function in the last decade, information of mTORC2 signaling is only rapidly expanding in recent years, from structural biology, signaling network, to functional impact. Here we review the recent advances in many aspects of the mTORC2 signaling, with particular focus on its involvement in the control of cell metabolism and its physiological implications in metabolic diseases and aging.

## 1. Introduction

**Figure 1:**
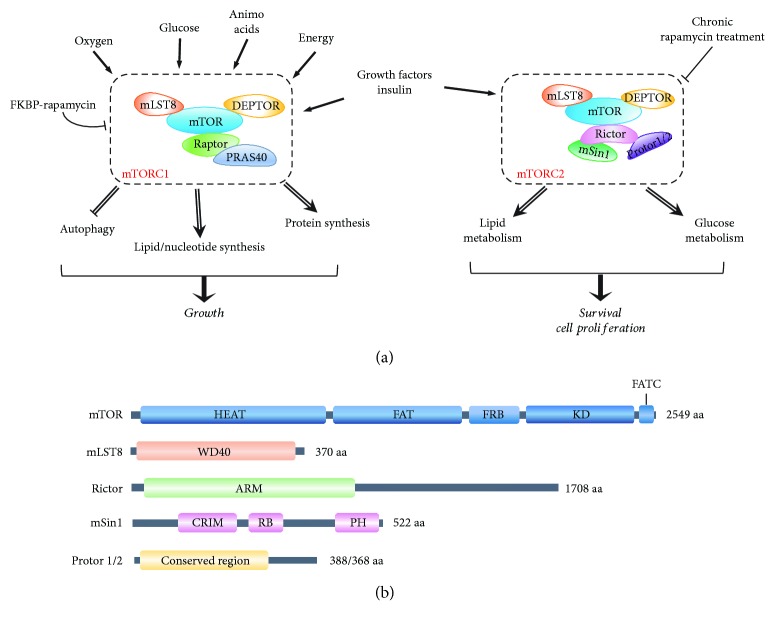
The protein composition of mTORC1 and mTORC2. (a) Schematic showing main molecular components and signals sensed by mTORC1 and mTORC2 (in the rectangles) and the processes they regulate to control cell growth and survival. With high sensitivity to rapamycin, mTORC1 senses oxygen, glucose, amino acids, energy, and growth factors to regulate cell growth by inhibiting autophagy and promoting several anabolic reactions, including synthesis of protein, lipids, and nucleotides. mTORC2 is insensitive to acute rapamycin treatment but responds to growth factors and insulin to regulate lipid and glucose metabolism, as well as survival and proliferation. (b) Schematic representation of mTORC2 core components. Domains of known function or structural motifs are indicated. Description of the abbreviations listed is contained within this review.

Mechanistic target of rapamycin (mTOR) is an evolutionarily conserved Ser/Thr kinase that belongs to the phosphatidylinositol-3-kinase-related kinase (PIKK) family. mTOR functions through two structurally and functionally distinct complexes, mTORC1 and mTORC2 [1]. Although the two mTOR complexes share three conserved subunits, mTOR, mLST8 (mammalian lethal with SEC13 protein 8, also known as G*β*L), and DEPTOR (DEP domain containing mTOR-interacting protein), they also contain complex-specific components. While mTORC1 comprises Raptor (regulatory-associated protein of mTOR) and PRAS40 (proline-rich Akt substrate of 40 kDa), mTORC2 distinctively contains Rictor (rapamycin-insensitive companion of mTOR) and mSin1 (mammalian stress-activated protein kinase-interacting protein 1) as well as Protors-1 and -2 (protein observed with Rictors-1 and -2, respectively) [2] (Figure 1(a)). In addition to this compositional difference, the two complexes also differ in their response to rapamycin with mTORC1 activity being acutely inhibited by rapamycin while mTORC2 only responding to long-term treatments [3].

mTOR signaling has been considered as a master regulator of homeostasis, which controls many anabolic and catabolic processes in response to nutrient availability [1]. This is especially well established in mTORC1 signaling through its regulation of anabolic process. Notably, recent studies with the utilization of new pharmacological tools and genetic models demonstrate that mTORC2 also plays fundamental roles in regulating cell metabolism [4].

In this review, we will summarize the recent advances in mTORC2 signaling, including the structure and function of its subunits, its upstream regulators, and downstream effectors. Particularly, the emphasis will be given to the current understanding of mTORC2 in cellular metabolism, including glucose, lipids, amino acids, nucleotides, and reactive oxygen species (ROS), and their impact in metabolic disorders and longevity.

## 2. The mTORC2 Signaling

### 2.1. Composition of mTORC2

mTORC2 is composed of several protein subunits, of which four are considered as core, essential components: mTOR and mLST8 shared in both mTOR complexes and Rictor and mSin1 specific to mTORC2 (Figure 1(b)).

mTOR is the catalytic subunit of the mTOR complexes, composed of over 2500 amino acids and evolutionarily conserved in eukaryotes, from yeast to human [1]. Structurally, the N-terminal half of the mTOR protein contains more than 30 tandem prototypical HEAT (Huntingtin, EF3A, ATM, and TOR) repeats forming two *α*-solenoids, followed by a FAT (FRAP, ATM, and TRRAP) domain and a FRB (FKBP12/rapamycin binding) domain, which is linked to the kinase domain, ending with the FATC (FAT C-terminus) domain at the C-terminus (Figure 1(b)) [5]. The kinase domain of mTOR has a bilobal structure that is in an intrinsically active conformation, with a catalytic mechanism remarkably similar to canonical protein kinases [6]. However, its active site is usually recessed due to the FRB domain and an inhibitory helix protruding from the catalytic cleft, indicating that its accessibility is strictly regulated [6]. This regulation points to substrate recruitment as a major mechanism controlling the kinase activity.

mLST8 is another highly conserved subunit shared between the two mTOR complexes [7]. The entire protein of 326 amino acids is composed of seven WD40 repeats, which fold into a *β*-propeller that generally serves as a scaffold for protein interactions. Although mLST8 stably binds to the kinase domain of mTOR in both mTOR complexes, it appears to differentially regulate the kinase activity in the two complexes. Although deletion of mLST8 has no clear effect on mTORC1 activity and integrity, its absence completely abolishes mTORC2 activity, indicating that it is essential for mTORC2 function, not mTORC1 [8].

Rictor is a defining member of mTORC2, containing over 1700 amino acids [3, 9]. Despite of its large size, no clearly identifiable domains or motifs can be mapped to it except several sections that are conserved from yeast to human [3]. Nonetheless, Rictor contains several armadillo-like helical repeat clusters in its N-terminal portion, which extensively interact with mTOR and have a profound impact on mTORC2 assembly [10, 11]. The importance of Rictor in mTORC2 is determined by the observation that depletion of Rictor disrupts mTORC2 assembly and activity, suggesting that it plays a vital role in the integrity and stabilization of mTORC2 [12].

mSin1, encoded by the *MAPKAP1* (mitogen-activated protein kinase-associated protein 1) gene, is another specific component of mTORC2, discovered by its interaction with Rictor [13, 14]. mSin1 is highly conserved in vertebrates and contains four possible domains (Figure 1(b)). The N-terminal TORC region is responsible for the interaction with mTOR and Rictor as its natural isoform without this region fails to constitute mTORC2 [14]. The C-terminal region contains a pleckstrin homology (PH) domain, which seems to be not necessarily required for mTORC2 assembly and activity but may function for its membrane localization [14]. The central region is the most conserved section and hence called conserved region in the middle (CRIM), which is linked to a RAS-binding (RB) domain at its C-terminal (Figure 1(b)). Structure analysis indicates that CRIM is an ubiquitin-like domain with a prominent acidic loop that is primarily responsible for the recruitment of mTORC2 substrates [15]. Therefore, mSin1 is considered to mainly function for substrate recruitment and selection, and the exact mechanisms, however, await for further investigation.

### 2.2. Structure of mTORC2

Human mTORC2 has an overall structural organization similar to that of mTORC1 and TORC2 of *Saccharomyces cerevisiae* [10, 11, 16]. It is a rhombohedron in shape formed by dimerization of two sets of the key subunits in the mTOR complexes, which are shaped in pseudo-2-fold symmetry and a prominent central cavity with the dimer of mTOR and mLST8 as a core [11]. In addition, the principal site for mTOR interacting with Rictor in this architecture is the same site occupied by Raptor in mTORC1, thereby sterically excluding Raptor incorporation in mTORC2. However, the current studies can only resolve the interaction between the N-terminal regions of mTOR and Rictor proteins in high resolution, whilst the interactions between the catalytic domain of mTOR and C-terminal part of Rictor and mSin1 have not been clearly determined due to their high flexibility and mobility [10, 11]. Although the structure of mTORC2 shares several features with that of mTORC1, the two complexes diverge remarkably in their overall size, the surface area of the protomer interface, the volume and shape of the central cavity, and, more importantly, the accessibility of the kinase domain. These differences are likely resulted from the distinct molecular compositions and have profound impact on the functions and regulations of the two complexes, rendering mTORC2 insensitive to rapamycin [11, 16]. The determination of the high-resolution mTORC2 architecture at its catalytic kinase domain is required to further decipher the structural organization, regulation, and activation of this important kinase.

### 2.3. Effectors of mTORC2

**Figure 2:**
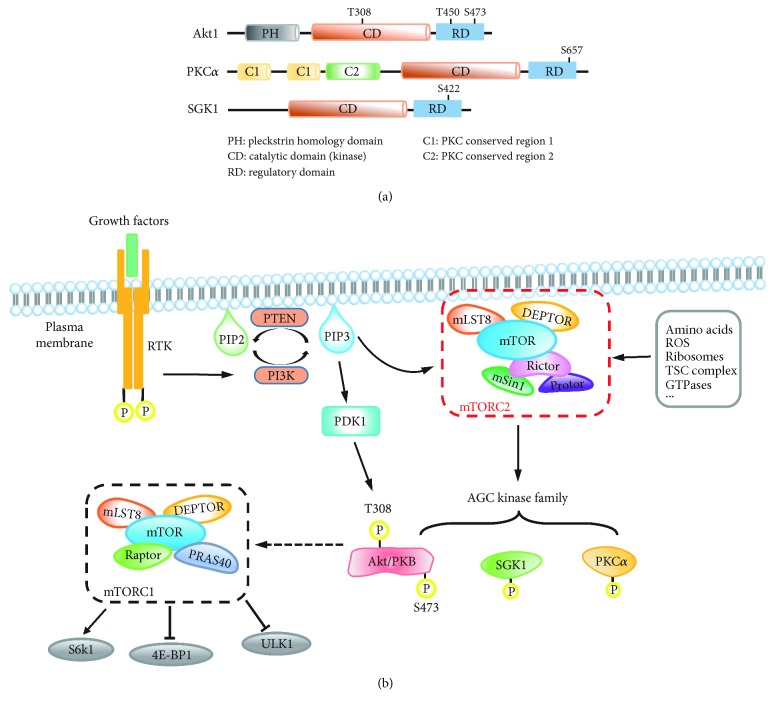
The signaling network of mTORC2. (a) Schematic representation of AGC kinases downstream of mTORC2. The major positions for phosphorylation are indicated. (b) As ligands, growth factors bind to the membrane receptor, receptor tyrosine kinase (RTK), which activates PI3K to phosphorylate PIP2 to PIP3 at the plasma membrane. PTEN (phosphatase and tensin homolog) dephosphorylates PIP3 and is a key negative regulator of PI3K signaling. PIP3 or other unknown factors activate mTORC2 in distinct manners to promote the phosphorylation of conserved motifs in several AGC kinases (Akt, PKC, and SGK1). For maximal activation, Akt is phosphorylated at T308 and S473 by PDK1 and mTORC2, respectively, and subsequently promotes the activation of mTORC1, which is characterized by phosphorylation of several downstream effectors, including S6K, 4E-BP1, and ULK1. There are several other upstream regulators which can also regulate mTORC2 activity, including amino acids, ROS, ribosome, TSC complex, and GTPases, through distinct mechanisms. Description of the abbreviations listed is contained within this review.

mTORC2 has a pleiotropic effect on cellular properties by means of activating/phosphorylating its downstream effectors, which are currently recognized to be mainly the members of the AGC kinase family, including PKB (protein kinase B, also called Akt), PKC (protein kinase C), and SGK1 (serum- and glucocorticoid-induced kinases 1) (Figure 2(a)) [17]. Upon activation by mTORC2, these AGC kinases also regulate multiple downstream targets, leading to cascading effects that impact on cell behavior and functions.

#### 2.3.1. Akt

Akt, a family of three members (Akt1, Akt2, and Akt3), is the most characterized effector of mTORC2 and a key factor downstream of PI3K signaling, which has important roles in cell survival, proliferation, and growth. Akt is located in cytosol in the absence of stimuli and is recruited to the plasma membrane via the interaction between its PH domain and phosphatidylinositol (3,4,5)-trisphosphate (PtdIns(3,4,5)P3) that is generated by PI3K activation. This leads to Akt phosphorylation at two residues, in the case of Akt1, T308 in the activation loop (A-loop) by PDK1 (phosphoinositide-dependent protein kinase 1) and S473 in hydrophobic motif (HM) by mTORC2 to ensure a full activation of Akt [18, 19]. Although Akt-S473 can also be phosphorylated by other kinases such as integrin-linked kinase (ILK) and DNA-dependent protein kinase (DNA-PK) in a cell/tissue-dependent manner [20, 21], it is predominantly phosphorylated by mTORC2 as deleting any of the mTORC2 key components results in dramatic abrogation of this phosphorylation [8, 12, 13]. Interestingly, although phosphorylation of both Akt-T308 and -S473 is required for a full activation of Akt, defective Akt-S473 phosphorylation affects only a subset of Akt targets *in vivo*, such as FoxO1/3a (forkhead box O1 and O3a), while other Akt targets, including TSC2 (tuberous sclerosis complex 2) and GSK3 (glycogen synthase kinase 3), and its downstream effector mTORC1 remain unaffected [13].

In addition to Akt-S473, mTORC2 has also been identified as the kinase for the phosphorylation of Akt-T450 in its turn motif (TM) to ensure proper folding and maturation of the Akt protein [22, 23]. Intriguingly, mTORC2 appears to phosphorylate Akt-T450 by a different mechanism from that of phosphorylating Akt-S473. Akt-S473 is phosphorylated by a canonical posttranslational mechanism and can be resembled by *in vitro* kinase assay with immunoprecipitated mTORC2, whereas Akt-T450 phosphorylation is thought to be performed by a cotranslational mechanism involving ribosomes and cannot simply be achieved by *in vitro* kinase assay [23, 24].

#### 2.3.2. PKC

PKC is a protein family of 11 members, which are categorized into three classes: the conventional (c) PKC (*α*, *β*1, *β*II, and *γ*), the novel (n) PKC (*δ*, *ε*, *θ*, and *η*/Λ), and the atypical (a) PKC (*ζ* and *ι*/*λ*) [25]. Both cPKCs and nPKCs are identified as mTORC2 substrates, being phosphorylated by mTORC2 at their HM and TM motifs [22, 23, 26, 27]. Among them, PKC*α* is the first identified and the most characterized substrate of mTORC2 and plays an important role in regulating actin cytoskeleton function, thereby affecting cell shape and mobility [3, 9]. However, the precise mechanisms by which mTORC2 phosphorylates PKC*α* remain ambiguous as neither HM nor TM phosphorylation of PKC*α* could be achieved by *in vivo* kinase assay with immunoprecipitated mTORC2 [23]. Furthermore, the functions of these phosphorylations in PKC*α* are also not very clear, but they have a role in protein stabilization and solubility, thus affecting PKC activity [28].

#### 2.3.3. SGK1

SGK1 is also phosphorylated and activated by mTORC2 at its S422 of HM, similar to that of Akt [29], and mSin1 has been shown to have a role in this phosphorylation, mediating the interaction between mTORC2 and SGK1 [30]. mTORC2-targeted SGK1 activation functions to regulate sodium transport as well as cell survival [31].

### 2.4. Regulation of mTORC2

The regulation of mTORC2 is much understudied and less understood than that of mTORC1 [1]. Nonetheless, increasing evidence in the last decade, particularly recent years, has emerged that mTORC2 activity could be affected by many factors via various mechanisms, which might directly or indirectly affect the localization, function, and stability of mTORC2 components as well as substrate availability.

#### 2.4.1. Membrane Localization and mTORC2 Activation

The plasma membrane plays an important role in mTORC2 activation, and a proportion of mTORC2 is located at the plasma membrane of mammalian cells to activate Akt [32, 33]. However, how mTORC2 is activated at the plasma membrane has been a mystery. It has been long thought that growth factors are the major signals to activate mTORC2 by means of the PI3K pathway (Figure 2(b)). Upon PI3K activation by insulin or other growth factors, PtdIns(4,5)P2 is phosphorylated to PtdIns(3,4,5)P3 to enable its binding to mSin1-PH, which subsequently releases the mSin1-PH inhibition on the mTOR kinase domain to activate mTORC2 activity. This interaction also enables the recruitment of mTORC2 to the plasma membrane where it meets and phosphorylates Akt that is also recruited to the plasma membrane via the Akt-PH domain [34]. However, a recent study, using biochemical labelling and imaging approaches, showed that the plasma membrane-associated mTORC2 is constitutively active and is independent of growth factor/PI3K signaling and that only the recruitment of substrate Akt to the plasma membrane requires the activation of PI3K [35]. Nevertheless, the mTORC2 activity at endosomes is sensitive to PI3K, suggesting the existence of spatially distinct mTORC2 subpopulations in response to growth factors. These studies raise further questions on whether and how environmental cues, such as growth factors, regulate mTORC2 activity.

#### 2.4.2. Component Modification and mTORC2 Activation

In response to upstream signals, mTORC2 components are susceptible to PTMs (posttranslational modifications), including phosphorylation, acetylation, and ubiquitination, which might serve to fine tune mTORC2 assembly, disassembly, activation, inactivation, or, perhaps, substrate recruitment.

The mTOR protein is phosphorylated at T2173 of the ATP-binding site in the kinase domain in an Akt-dependent manner, which impairs mTORC2 activity, thus functioning as a negative feedback to control mTORC2 activity [36]. The mTOR protein is also phosphorylated at S2481, and this phosphorylation is shown to depend on the intactness of mTORC2 activity but is unclear about its function, which is thus mainly used as a marker of mTORC2 [37]. The phosphorylation of mTOR at S2448 is thought to be associated with mTORC1 rather than mTORC2 [37].

The mTORC2 activity is also regulated by ubiquitination of mLST8 and DEPTOR. The K63-linked polyubiquitination of mLST8 by TRAF2 E3 ubiquitin ligase disrupts mTORC2 formation, thereby reducing mTORC2 activity. In contrast, deubiquitination of mLST8 by OTU domain-containing protein 7B (OTUD7B) deubiquitinase promotes its interaction with mSin1, facilitating the formation of mTORC2 and enhancing its activity [38]. DEPTOR, an inhibitory subunit of both mTOR complexes, is phosphorylated by mTOR and casein kinase 1 (CK1) in response to growth factor stimulation, which results in its degradation and mTOR activation [39–41].

Moreover, the mTORC2 scaffold protein Rictor also contains multiple modifiable sites, which might impact on mTORC2 activity. Rictor-T1135 has been found phosphorylated by S6K1, but whether this phosphorylation has any effect on mTORC2 activity remains controversial [42, 43]. In addition, Rictor has also been reported to be phosphorylated by GSK3 at S1235 or T1695. Although both appear to negatively affect mTORC2 activity, they function through distinct mechanisms [44, 45]. The phosphorylation of Rictor-S1235 interferes with the interaction between mTORC2 and the substrate Akt, whilst the phosphorylation of Rictor-T1695 promotes FBXW7-mediated degradation of Rictor [44, 45]. Furthermore, acetylation of Rictor at multiple sites between residues 1040–1140 has been reported to activate mTORC2 [46, 47].

Phosphorylation of mSin1 has also been shown to have an effect mTORC2 activity but with disputed results. In one report, mSin1-T86/T398 were shown to be phosphorylated by S6K or Akt, resulting in inhibited mTORC2 activity [48], whilst in other reports, Akt-mediated mSin1-T86 phosphorylation was shown to enhance mTORC2 [49, 50]. The reason for this discrepancy remains unclear. In addition, mSin1-S260 was shown to be phosphorylated by mTOR under energy-sufficient condition to stabilize mSin1, thus enhancing mTORC2 activity [51].

#### 2.4.3. Other Factors

In addition to the above regulators, some other factors are also emerging to have a role in the regulation of mTORC2 activity, such as ribosomes, small GTPases, TSC complex, and amino acids (AAs). It is revealed that mTORC2 activity requires well-assembled intact ribosomes and that the insulin-stimulated PI3K pathway promotes their physical interaction [52]. The involvement of ribosomes in mTORC2 activation is supported by the finding of cotranslational phosphorylation of Akt-T450 [24]. Therefore, ribosomes might serve as a scaffolding platform that facilitates mTORC2 to phosphorylate some of its targets. However, the precise mechanism by which mTORC2 is activated by association with ribosomes remains to be elucidated. TSC complex and small GTPases (e.g., Rhy1, Rit, and Rac1) have been demonstrated to positively regulate mTORC2 activity by physically interacting with mTORC2 components [53–55]. Moreover, mTORC2 activity can be activated by glutamine depletion mediated by Sestrin2 with an unestablished underlying mechanism [54, 56].

## 3. mTORC2 in Cell Metabolism

### 3.1. Glucose Metabolism

**Figure 3:**
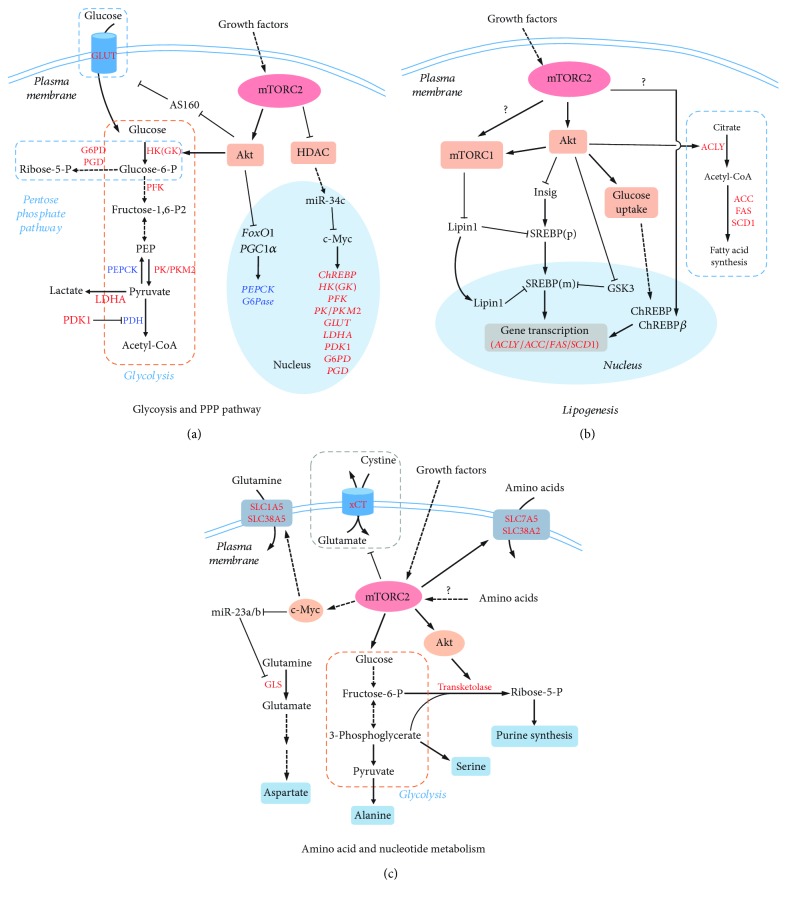
mTORC2 in cell metabolism. (a) mTORC2 promotes glucose metabolism via glycolysis and PPP (pentose phosphate pathway). In response to growth factors, mTORC2 activates glucose catabolism through two main factors, Akt and c-Myc. Akt activates glycolysis at both transcriptional and posttranslational levels. c-Myc enhances the expression of genes involved in glycolysis and PPP. Activated factors are shown in red letters; suppressed factors are shown in blue. Solid line indicates direct regulation; dash line indicates indirect regulation. (b) mTORC2 promotes lipogenesis via Akt-dependent and -independent mechanisms. mTORC2 activates, via Akt, SREBP and ChREBP, two transcription factors for the expression of lipogenic genes, such as ACLY, ACC, FAS, and SCD1. mTORC2 may also stimulate lipogenesis by activating mTORC1 in an Akt-independent manner. (c) mTORC2 regulates amino acids and nucleotide metabolism. mTORC2 regulates amino acid transport by modulating activity of amino acid transporters, SLC7A5 and SLC38A2, and antiporter, xCT. In addition, mTORC2 activates glutamine transporters, SLC1A5 and SLC38A5, via c-Myc to promote glutamine uptake. By activating glycolysis, mTORC2 increases the production of 3-phospholycerate and pyruvate, which can be used to synthesize serine and alanine. mTORC2 promotes glutaminolysis via c-Myc, which transforms glutamine to glutamate and aspartate. Purine synthesis can be stimulated by mTORC2 through Akt-mediated activation of transketolase. Description of the abbreviations listed is contained within this review.

Emerging evidence suggest that mTORC2 plays an important role in glucose metabolism and homeostasis (Figure 3(a)). mTORC2 signaling affects many aspects of glucose metabolism, including glucose uptake, glycolysis, gluconeogenesis, and oxidative phosphorylation [57–63].

#### 3.1.1. Glucose Uptake

The regulation of glucose uptake is a key determinant of glucose metabolism [64]. Tissue-specific Rictor-knockout mice exhibit compromised glucose uptake in the liver, adipose tissue, and muscle while the underlying mechanisms seem to be divergent and tissue type dependent [58–61]. In the muscle, deletion of Rictor results in the failure of Akt-mediated AS160 phosphorylation in response to insulin stimulation, which subsequently decreases the plasma translocation of GLUT4, a glucose transporter [59]. In the liver, the decreased glucose uptake in Rictor-null cells is mainly resulted from a reduction in the expression and activity of glycolysis rate-limiting enzyme glucokinase (GK) [61]. In adipose tissue, mTORC2 contributes to glucose uptake mainly by regulating either hexokinase (HK, isoenzyme of GK) or GLUT4 [57, 58, 60]. In brown adipose tissue (BAT), mTORC2 activates cytosolic HK activity through an Akt-S473 phosphorylation-associated mechanism, which has no effect on GLUT1 and GLUT4 translocation [60], while, in white adipose tissue (WAT), mTORC2 stimulates the plasma membrane translocation and gene expression of a glucose transporter, GLUT1 or GLUT4, in an Akt-independent manner [58, 60].

#### 3.1.2. Glycolysis

mTORC2 also regulates glucose metabolism by facilitating glycolysis. In Rictor-knockout liver, glycolysis is significantly reduced due to the downregulation of glycolytic genes, including GK, liver-type pyruvate kinase (L-PK), and carbohydrate response element-binding protein (ChREBP), and the decreased enzymatic activity of GK [61]. However, this Rictor deficiency seems to have no effect on overall glycolysis in the whole body [63]. In contrast, adipose-specific Rictor ablation causes decreased glycolysis in fat as well as in the whole body, possibly resulting from decreased HK activity [57, 58, 60]. mTORC2 is able to increase HK activity and glycolysis via Akt-dependent and -independent mechanisms [62]. In glioblastoma, mTORC2 upregulates the expression of glycolytic genes, including HK, by increasing the levels of transcription factor c-Myc. Mechanistically, mTORC2 phosphorylates and inactivates HDAC (histone deacetylase), leading to the increase of FoxO1 and FoxO3 acetylation. The acetylation in FoxOs attenuates their transcriptional activity and reduces the expression of miR-34-c that suppresses c-Myc expression [65]. Therefore, mTORC2 may also promote glycolysis through multiple mechanisms depending on tissue/cell types.

#### 3.1.3. Gluconeogenesis

Gluconeogenesis is a glucose production process that utilizes pyruvate to produce glucose. Although sharing several enzymes with glycolysis implements reversible reactions, gluconeogenesis also has its specific enzymes that catalyze key irreversible steps (Figure 3(a)). For example, phosphoenolpyruvate carboxykinase (PEPCK) catalyzes the formation of phosphoenolpyruvate (PEP); fructose 1,6-bisphosphatase converts fructose 1,6-bisphosphatase to fructose 6-phosphate and glucose-6-phosphatase (G6Pase), finally turning glucose 6-phosphate to glucose. Gluconeogenesis is important for maintaining blood glucose levels during starvation and is reciprocally regulated with glycolysis [66]. In contrast to glycolysis, mTORC2 functions to suppress gluconeogenesis, and therefore, in the absence of Rictor, the mice exhibit hyperglycemia due to increased gluconeogenesis [58, 61, 63]. The effect of mTORC2 on gluconeogenesis mainly results from the upregulation of several key gluconeogenic enzymes, such as G6Pase and PEPCK, through Akt-mediated FoxO1-phosphorylation, which leads to nuclear exclusion of FoxO1 and reduction of its transcriptional activities [67, 68]. In addition, mTORC2-mediated Akt activation is also responsible for the phosphorylation and inhibition of PGC1*α* (peroxisome proliferator-activated receptor gamma coactivator 1-alpha), a transcription coactivator involved in activating gluconeogenic gene expression [69].

### 3.1.4. Oxidative Phosphorylation

It is noteworthy that both mTORC2 effectors of Akt and c-Myc have inhibitory effect on oxidative phosphorylation via activation of HK2 and pyruvate dehydrogenase kinase 1 (PDK1), respectively [62, 70, 71]. Indeed, Rictor-deficient cells and mice exhibit elevated mitochondrial respiration. Moreover, activated Akt signaling stimulates the oxidative to glycolytic metabolic shift in the muscle [72].

Taken together, mTORC2 regulates glucose metabolism by promoting glucose uptake and glycolysis and inhibiting gluconeogenesis and oxidative phosphorylation.

### 3.2. Lipid Metabolism

Increasing evidence demonstrates that mTORC2 also plays a pivotal role in lipid metabolism by increasing lipogenesis while suppressing lipolysis and fatty acid *β*-oxidation (Figure 3(b)) [73]. In liver-specific Rictor-knockout mice, both hepatic and serum triglyceride levels exhibited a reduction [61], while in adipose-specific Rictor-knockout mice, only serum triglycerides were decreased [57, 58]. Nonetheless, both knockout strains showed altered lipid composition and metabolism [57, 58, 61].

#### 3.2.1. Lipogenesis

Rictor deficiency results in reduced lipogenesis, which is not only due to decreased glucose uptake (discussed above) but also due to decreased expression of genes encoding lipogenic enzymes, such as acetyl-CoA carboxylase (ACC), ATP-citrate lyase (ACL), fatty acid synthase (FAS), and stearoyl-CoA desaturase (SCD1) [58, 61, 63, 74]. Two transcription factors, ChREBP and SREBP1c (sterol regulatory element-binding protein 1c), regulate the expression of these key lipogenic enzymes [75, 76], and mTORC2 has been reported recently to be an upstream regulator of ChREBP and SREBP1c and therefore lipogenesis [58, 61].

In adipose-specific Rictor-knockout mice, ChREBP expression is decreased, and this reduction can be rescued by restoration of glucose uptake and glycolysis, suggesting that mTORC2 positively regulates ChREBP expression through modulating glucose flux and metabolism [77, 78]. ChREBP protein can undergo multiple different posttranslational modifications, which can impact on its nuclear localization and transcriptional activity [78]. It is thought that high glucose concentration leads to the dephosphorylation of ChREBP, enabling its nuclear entry, thus enhancing its transcriptional activity. Furthermore, ChREBP has two isoforms from alternative promoters, a canonical abundant *α* isoform and a novel *β* isoform. Glucose flux activates the *α* isoform, which induces the expression of the less abundant but more active *β* isoform to activate the gene expression of lipogenic enzymes [77]. Interestingly, Rictor*-*knockout adipose tissue exhibits reduced ChREBP-*β* expression without altering ChREBP-*α* protein levels [58], implying that mTORC2 affect ChREBP expression and activity probably through affecting ChREBP-*α* protein modification.

Unlike ChREBP, mTORC2-mediated SREBP1c activation is through a glucose uptake-independent mechanism [61, 74]. In both liver-specific and *Myf5* lineage-specific Rictor-knockout mice, it showed reduced expression of SREBP1c, which may account for the changes detected in lipogenesis [61, 74]. In both studies, downregulation of SREBP1c expression by mTORC2 deficiency appeared to be associated with the defect in Akt-S473 phosphorylation as expression of Akt-S473D could rescue the expression in SREBP1c and the phenotype in lipogenesis [61, 74]. However, another study argued that mTORC2-mediated regulation of SREBP1c is through an Akt-independent mechanism [63]. Therefore, this discrepancy might require further studies to clarify.

The pentose phosphate pathway (PPP) is a metabolic pathway parallel to glycolysis and is considered as a major source of NADPH (nicotinamide adenine dinucleotide phosphate), which is required for reducing power during de novo lipid biosynthesis [79]. mTORC2 is involved in the regulation of PPP similar to that of glycolysis by promoting the expression of G6PD and PGD as discussed above. Therefore, it is likely that the connection between mTORC2 and PPP might contribute to lipogenesis and anabolic cell growth and proliferation.

#### 3.2.2. Lipolysis and Fatty Acid *β*-Oxidation

In consistence with its positive effect on lipogenesis, mTORC2 suppresses lipolysis and fatty acid *β*-oxidation. Rictor-deficient mice exhibit increased serum glycerol and free fatty acids (FFA), which possibly resulted from the sustained activation of protein kinase A- (PKA-) dependent lipolysis, and the elevated expression of fatty acid oxidation-related genes, including acyl-CoA oxidase (ACO), carnitine palmitoyltransferase 1 (CPT1), and peroxisome proliferator-activated receptor alpha (PPAR*α*) [57, 58, 61, 80]. Although elevated FFA is not necessarily associated with insulin resistance [81], increasing adipose lipolysis could increase hepatic glucose production and cause insulin resistance [82].

### 3.3. Other Metabolisms

#### 3.3.1. Amino Acid Metabolism

Amino acids are essential nutrients as the building block of proteins and carbon/nitrogen sources of many metabolic pathways. Increasing evidence has emerged that mTORC2 is an important regulator participating in the amino acid metabolism (Figure 3(c)).

In response to insulin or EGF (epidermal growth factor) stimulation, mTORC2 regulates amino acid uptake by increasing FoxO-dependent c-Myc transcription and translation, which in turn upregulates the expression of glutamine transporters, SLC1A5 (SN1) and SLC38A5 (SN2) [65]. In addition, mTORC2 could also regulate amino acid metabolism by posttranslationally altering transporter/antiporter activity [83, 84]. For example, mTORC2 regulates cell surface abundance of specific transporter isoforms SLC7A5 and SLC38A2 without affecting their global protein expression [84], and mTORC2 phosphorylates of serine 26 and inhibits cystine-glutamate antiporter xCT-mediated cystine uptake and glutathione synthesis [83]. Moreover, mTORC2 can stimulate glutamine metabolism via c-Myc. c-Myc transcriptionally represses miR-23a and miR-23b, resulting in greater expression of mitochondrial glutaminase, thereby upregulating glutaminolysis [65, 71, 85]. Increased glutaminolysis can provide *α*-ketoglutarate and amidogen for the synthesis of nonessential amino acids [86]. Similarly, mTORC2 promotes the generation of pyruvate and 3-phosphoglycerate through glycolysis, which would provide carbon skeleton for nonessential amino acid synthesis [87]. Although speculative, mTORC2 inhibits autophagic and proteasomal protein turnover [88], the alteration of which may also participate in amino acid homeostasis [89].

#### 3.3.2. Nucleotide Metabolism

mTORC2-activated glucose metabolism can provide nucleotide synthesis with essential materials [90]. First, mTORC2 stimulates glycolysis and thus supplies 3-phosphoglycerate and pyruvate, which serve as the precursors of serine, glycine, aspartate, glutamate, and glutamine synthesis. Along with CO_2_ and ATP, glycolysis provides the carbon/nitrogen units and energy for both purine and pyrimidine de novo synthesis. Second, mTORC2 directs glucose flux into the pentose phosphate pathway (both oxidative and nonoxidative phases). This guarantees the sufficient production of ribose-5-phosphate and thus phosphoribosyl pyrophosphate (PRPP), which forms the ribose unit of the nucleotide. In addition, the pentose phosphate pathway gives rise to an abundant pool of NADPH as the reducing power for desoxyribonucleic acid production. Consistently, a systematic translational analysis reveals that Rictor protein accumulates significantly during the S phase of the cell cycle rather than G1 and mitosis [91]. This suggests that mTORC2 may play an important role in providing dNTPs for DNA synthesis during the S phase.

mTORC2 positively regulates transketolase activity for purine synthesis via Akt-mediated phosphorylation [92]. Transketolase catalyzes the formation of ribose-5-phosphate from the nonoxidative pentose phosphate pathway, which is then transformed to PRPP for purine synthesis. It is of great importance that this discovery mechanistically links mTORC2-mediated glucose metabolism and nucleotide synthesis. This implies that mTORC2 contributes to nucleotide synthesis via providing essential substrates. Moreover, essential amino acids are indispensable for the activation of mTORC2 and purine synthesis. This also indicates that the existence of essential amino acids may manifest an energy-sufficient state so that cells can put lots of energy and materials into nucleotide production. Recent studies reveal the mechanism of how mTORC1 is involved in nucleotide synthesis [93]. The two mTOR complexes may coordinate to facilitate nucleotide synthesis.

#### 3.3.3. ROS Metabolism

Aside from being activated by ROS, mTORC2 is, in turn, involved in ROS metabolism by regulating GSH (glutathione) and NADPH synthesis [71]. GSH pool is the important cellular redox buffer to protect the cell from oxidative damage, and NADPH can maintain the GSH in the reduction state. At the cost of glutamine, lowered mTORC2 activity facilitates cystine uptake via cystine-glutamate antiporter, which is then incorporated into GSH [83]. Although paradoxical, mTORC2 can regulate cellular content of glutamate, glycine, and cysteine, which form GSH. As for NADPH, mTORC2 facilitates its production through the pentose phosphate pathway. However, except for the antioxidant, it is not clear whether ROS scavenging enzymes are also under regulation of mTORC2. Even though the relationship between mTORC2 and ROS metabolism remains unclear, evidence is gathering, and the prospect is promising.

## 4. Impact of mTORC2-Mediated Metabolism on Diseases

**Figure 4:**
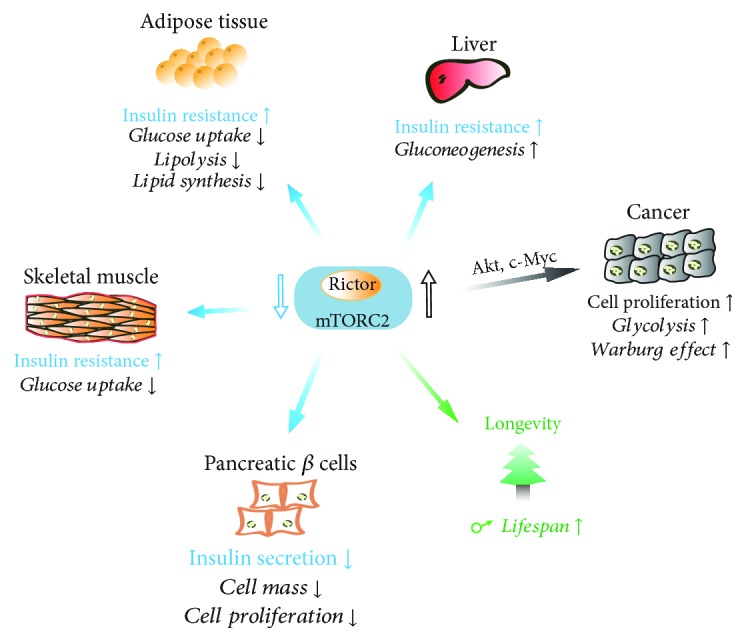
Impact of mTORC2-mediated metabolism on T2DM, cancer, and aging. In T2DM, the suppression of mTORC2 leads to gluconeogenesis in the liver and impaired glucose uptake in the muscle and adipose tissue, leading to insulin resistance. In pancreatic *β* cells, mTORC2 dysfunction also leads to reduced *β*-cell mass, proliferation, and impaired insulin secretion. In many types of cancers, mTORC2 activation promotes glucose uptake and glycolysis, which may contribute to the altered glucose metabolism and Warburg Effect, which fuels cell proliferation. In mammals, mTORC2 activity promotes longevity in males without established mechanism.

mTORC2 signaling plays an important role in metabolic regulation, the dysregulation of which is closely related to human diseases, including T2DM (type 2 diabetes mellitus), cancer, and aging (Figure 4).

### 4.1. T2DM

T2DM is a metabolic disorder, characterized by dysregulation of carbohydrate, lipid, and protein metabolism as a result of impaired insulin secretion, insulin resistance, or both combined [94]. Insulin resistance, a fundamental mechanism causing T2DM, results in disturbed glucose homeostasis with increased glucose production in the liver and decreased glucose uptake in the muscle and adipose tissue. As a convergent node in insulin signaling cascade and a master regulator of homeostasis, deregulation of mTOR signaling, including mTORC2, impairs insulin signal transduction and its biological actions, leading to metabolic disorders, including T2DM. This has been clearly demonstrated in the studies of Rictor-knockout mice. Although germ-line deletion of Rictor in mice resulted in embryonic lethal at mid-gestation probably due to defects in placenta and fetal vascular development [8, 95], mice with tissue-specific Rictor-knockout in the liver, muscle, or adipose tissue all exhibited severe insulin resistance [57, 59, 61, 63]. This could be attributed to the fact that mTORC2 suppresses gluconeogenesis in the liver while promoting glucose uptake in the muscle and adipose tissue as discussed above. Therefore, disturbance in mTORC2 signaling may underlie the abnormality of glucose metabolism in T2DM, leading to hyperglycemia. Furthermore, mTORC2 could also contribute to T2DM through acting on pancreatic *β* cells as many mTORC2 downstream targets, including Akt, PKC*α*, FoxO1/3, and MST1 (mammalian sterile 20-like kinase 1), are essential for *β*-cell survival and insulin production [96]. Indeed, it has been reported that patients with T2DM have dramatically reduced mTORC2 activity in pancreatic *β* cells [97]. Therefore, mTORC2 is very important for maintaining overall glucose metabolism and homeostasis in response to nutrient fluctuations and metabolic demand.

In addition to glucose, lipid metabolism is also altered in T2DM. Independent studies suggest that adipose tissue-specific knockout of Rictor leads to decreased lipogenesis and increased lipolysis in adipose tissue, which appear to result in hepatic insulin resistance and hepatic steatosis in these mice [57, 58, 80]. The dysregulation of lipid metabolism in adipose tissue has also been associated with insulin resistance in humans [98]. Mechanistically, fat-specific ablation of Rictor may contribute to insulin resistance through multiple ways. First, Rictor deficiency in adipose tissue reduces the glucose uptake in this tissue, leading to a shortage of glucose supply, which reduces lipogenesis and increases the risk of hepatic insulin resistance [77, 99]. Second, it also increases lipolysis, leading to increased circulating FFAs, which then accumulate in tissues and impair insulin signaling [100]. Finally, Rictor ablation causes decreased lipid synthesis and altered lipid composition in adipose tissue, which may disrupt the synthesis of active lipid to improve insulin sensitivity [101].

### 4.2. Cancer

Deregulation of mTORC2, particularly hyperactivation, has been commonly observed in many types of human cancers. Mutations and aberrant amplifications of mTORC2 core components are two main factors contributing to its hyperactivation. For example, mutations in the mTOR-FAT domain decrease mTOR binding to the inhibitor DEPTOR, thereby conferring the hyperactivation of both mTOR complexes [102]. In addition, Rictor has also been identified to be highly mutated [102] and abnormally overexpressed through genetic and epigenetic regulations [103] in a variety of cancer types. The exact functions of these abnormalities in tumorigenesis and treatment await identification. Nonetheless, mTORC2 regulates AGC kinase family proteins, such as Akt and PKC, for their stabilization and activity, which have important roles in the cell proliferation, survival, and migration, thereby having a crucial role in cancer [2].

In terms of metabolism, mTORC2 activation promotes glucose uptake, facilitates glycolysis, and inhibits oxidative phosphorylation, which may contribute greatly to the alteration of glucose metabolism in cancer cells, known as the Warburg Effect, conferring to a high rate of cell proliferation [71, 79]. Furthermore, mTORC2-mediated lipogenesis is identified to promote hepatocellular carcinoma, particularly by stimulating sphingolipid and glycerophospholipid synthesis, which fuels cancer cell growth and energy production [104].

In addition, Rapamycin derivatives have been applied to cancer treatment in clinical trials with limited efficiency, which is thought to be due to the limitation to inhibit mTORC2 [2]. As such, the second-generation ATP-competitive inhibitors against mTOR kinase have entered clinical trials [105]. These mTOR kinase inhibitors show greater inhibitory effects on both complexes and are more effective in inhibiting cancer cell growth [106]. Moreover, dual mTOR/PI3K kinase inhibitors have been developed in order to fully suppress Akt and 4EBP1 activation [107]. Recently, selective inhibition of mTORC2 signaling by a nanoparticle-based RNAi therapy showed being able to effectively block breast cancer cell growth and survival [108]. All these suggest that mTORC2 might be a good therapeutic target for cancer treatment.

### 4.3. Aging

Aging is a natural process, commonly accompanied by a progressive loss of physiological functions and increased susceptibility to age-associated diseases, including cardiovascular diseases, neurodegenerative diseases, infectious diseases, and cancers, thus ultimately limiting the health and lifespan [109]. The velocity of aging is affected by a large number of environmental or genetic factors, such as nutrition sensing, DNA damage, stem cell maintenance, energy, and oxidative metabolism [110], and three sets of key proteins have been reported to modulate aging process: mTOR signaling, insulin/IGF (insulin-like growth factor) signaling (IIS), and sirtuin family proteins [111]. Using genetic or pharmacological intervention, mTOR was discovered as a regulator of lifespan in *Caenorhabditis elegans* [112], *Drosophila melanogaster* [113], and *Saccharomyces cerevisiae* [114] as well as mice [115]. Inhibition of mTOR by rapamycin showed 9–14% increase in the maximum lifespan of both male and female mice [115], indicating the importance of mTOR signaling in longevity.

Given the long administration of rapamycin in these mice, its effect could be the inhibition of both mTOR pathways, suggesting that mTORC2 might also have a role in aging. However, exact functions of mTORC2 remain unknown. In a separate study using mouse models in which Rictor has been reduced or deleted conditionally [116], it showed that the lifespan is significantly decreased in males with deficient Rictor but not in females and that the deleterious effect on lifespan was independent of the glucose intolerance resulting from Rictor deletion. Other studies of longevity on mTORC2 substrates yield conflicting results. In *Caenorhabditis elegans*, mTORC2-SGK1 activation affects the lifespan through two pathways and generates opposing outcomes depending on the cellular and environmental contexts [117]. In mice with heterozygous Akt1, it showed an increase in the lifespan [118].

Taken together, it suggests that mTORC2 signaling has important functions in regulating aging and aging-associated diseases, but the roles they play are complex and influenced by other factors.

## 5. Conclusion and Perspective

With the exciting progress accomplished over the last few years, it becomes clearer than before that mTORC2 signaling plays vital roles in tissue homeostasis and human health. These are implemented by its function as a crucial signaling hub, in response to both environmental and intracellular changes, to regulate several signaling networks and many essential cellular functions, such as cell proliferation, survival, migration, and metabolisms. However, our understanding of mTORC2 is far from complete. There are still several important questions awaiting to be addressed.

### 5.1. mTORC2 Localization and Structure

Unlike mTORC1, mTORC2 have been observed to localize at several distinct membranous compartments, e.g., plasma membrane, mitochondria, ER (endoplasmic reticulum), and MAM (mitochondria-associated ER membrane) [119]. However, why mTORC2 have so many subcellular distributions and whether mTORC2 at different compartments sense different inputs and regulate distinct substrates remain unclear. It is possible that mTORC2 utilizes various subcellular localizations to different membranous structures as a general principle to enable its spatiotemporal activation by diverse signals and mechanisms.

In addition, it remains incomplete in our understanding of the mTORC2 structure, particularly association between the mTOR kinase domain and other components of mTORC2, such as Rictor and mSin1 [11, 120]. A better elucidation of mTORC2 structure on this crucial part might aid us to determine the molecular mechanisms by which it is regulated and functions on its substrates.

### 5.2. Regulation of mTORC2

Currently, the regulatory mechanism governing mTORC2 activation is fairly controversial. Although growth factors have been recognized as the major activating signal, little is known about how exactly they control mTORC2 [34, 35]. Moreover, does mTORC2 respond to other environmental cues in addition to growth factors? For example, the activation of TORC2 in yeast is not responsive to growth factors, and instead, the lipid binding is a crucial requirement [121]. The ligand engagement of Toll-like receptor (TLR) is also able to activate mTORC2 [122, 123]. Furthermore, mTORC2 that localizes at the plasma membrane is constitutively active in mammalian cells [35]. In this context, could nutrients, cellular stress, or other metabolic signals generated from the plasma membrane be the primary regulator of mTORC2? The identification of upstream regulators of mTORC2 might provide novel insights into these uncertainties.

It should also be noted that several mTORC2 components undergo PTMs, including phosphorylation, ubiquitination, acetylation, and palmitoylation, to regulate mTORC2 stability and assembly. mTORC2 signaling network is so intricately intertwined with multiple signal and metabolic pathways. Crosstalk between these pathways may extensively modulate mTORC2 [124].

### 5.3. Targets of mTORC2 Signaling

Although it has long been regarded that mTORC2 exerts wide spectrum effects on cellular properties through members of the AGC kinase family, the exact mechanisms for mTORC2 governing their activation are incompletely understood. For example, why does mTORC2 phosphorylate Akt-HM and -TM through different mechanisms, and is ribosome required for both? Why is the immunoprecipitated mTORC2 only able to phosphorylate the Akt-HM but not the PKCa-HM in *in vitro* kinase assays? Moreover, many proteins, in addition to the three AGC kinases, have been found to be modified in a mTORC2-dependent manner, such as HDAC [65], ChREBP-*α* [58], and Smad2/3 [125]. However, it is unclear whether this is a direct or indirect modification as it is largely unknown the mechanism by which mTORC2 recruits its substrates, which might depend on the localization of mTORC2 and may also involve other factors.

Finding answers to these questions will greatly expand our knowledge on mTORC2 biology, aiding the design and development of mTORC2-specific agonists and inhibitors, which will also help the development of therapeutic avenues to treat human metabolic diseases. The future will undoubtedly continue to bring unexpected insight on this remarkable pathway.
